# HCV and HCC Tango—Deciphering the Intricate Dance of Disease: A Review Article

**DOI:** 10.3390/ijms242216048

**Published:** 2023-11-07

**Authors:** Ivana Milosevic, Nevena Todorovic, Ana Filipovic, Jelena Simic, Marko Markovic, Olja Stevanovic, Jovan Malinic, Natasa Katanic, Nikola Mitrovic, Natasa Nikolic

**Affiliations:** 1 Faculty of Medicine, Department for Infectious Diseases, University of Belgrade, 11000 Belgrade, Serbia; ivana.milosevic@med.bg.ac.rs (I.M.); markovicdrmarko@gmail.com (M.M.); stevanovicolja74@gmail.com (O.S.); jovan.malinic@gmail.com (J.M.); nikolabmi@gmail.com (N.M.); 2 University Clinic for Infectious and Tropical Diseases, University Clinical Center of Serbia, Bulevar Oslobodjenja 16, 11000 Belgrade, Serbia; nevena.todorovic.1992@gmail.com (N.T.); anafilipovic0211@gmail.com (A.F.); simicj093@gmail.com (J.S.); katanicn@gmail.com (N.K.); 3 Faculty of Medicine, University of Pristina Situated in Kosovska Mitrovica, 28000 Kosovska Mitrovica, Serbia

**Keywords:** hepatitis C virus, hepatocellular carcinoma, oncogenesis, epigenetic changes, immune dysregulation

## Abstract

Hepatitis C virus (HCV) is a major cause of hepatocellular carcinoma (HCC) accounting for around one-third of all HCC cases. Prolonged inflammation in chronic hepatitis C (CHC), maintained through a variety of pro- and anti-inflammatory mediators, is one of the aspects of carcinogenesis, followed by mitochondrial dysfunction and oxidative stress. Immune response dysfunction including the innate and adaptive immunity also plays a role in the development, as well as in the recurrence of HCC after treatment. Some of the tumor suppressor genes inhibited by the HCV proteins are p53, p73, and retinoblastoma 1. Mutations in the telomerase reverse transcriptase promoter and the oncogene catenin beta 1 are two more important carcinogenic signaling pathways in HCC associated with HCV. Furthermore, in HCV-related HCC, numerous tumor suppressor and seven oncogenic genes are dysregulated by epigenetic changes. Epigenetic regulation of gene expression is considered as a lasting “epigenetic memory”, suggesting that HCV-induced changes persist and are associated with liver carcinogenesis even after cure. Epigenetic changes and immune response dysfunction are recognized targets for potential therapy of HCC.

## 1. Introduction

Hepatocellular carcinoma (HCC) is the most common type of primary liver cancer, accounting for up to 90% of cases. Primary liver cancer is the sixth most frequent cancer in the world, with over 800,000 new cases each year. It stands as one of the major contributors to cancer-related deaths in men, responsible for 745,000 annual fatalities [1]. HCC has been recognized as the cause of death in 54–70% of patients with compensated cirrhosis of different etiologies [2]. It is expected that there will be an increase in incidence after 2025, with more than 1 million cases on an annual basis [1]. HCC in most cases arises from chronic liver disease caused by hepatitis B virus (HBV), hepatitis C virus (HCV), or nonalcoholic steatohepatitis (NASH)—according to the new nomenclature metabolic dysfunction-associated steatohepatitis (MASH). Diabetes and obesity are additional risk factors for the development of this neoplasm [3].

About 60–70% of HCC cases have been associated with HBV or HCV infection [4]. The main carcinogenetic factor is HBV, but due to the compulsory vaccination against HBV and the use of suppressive antiviral therapies in patients with chronic hepatitis, the incidence of HBV as an etiological factor is slowly decreasing. HCV is also a major cause of HCC and one-third of HCC cases have been reported to be caused by hepatitis C [5]. It is estimated that chronic hepatitis C (CHC) occurs in 55–85% of people infected with HCV, and 20–30% of people with CHC acquire liver failure or cirrhosis [6]. The data show that the annual incidence of HCC in patients with HCV and cirrhosis is approximately 1–4%. Apart from developing in patients with advanced fibrosis and cirrhosis, it is interesting that over the course of 30 years, 1–3% of patients with HCV without cirrhosis will also eventually develop HCC [7,8]. Due to the introduction of direct-acting antiviral (DAA) drugs, which enable the eradication of HCV, it is expected that the incidence of HCC will decrease in the future. Nevertheless, the issue of the occurrence and recurrence of HCC in patients who have successfully eliminated HCV remains a topic requiring further discussion [9].

## 2. HCV Infection and Development of HCC—Key Aspects of Pathogenesis

The pathogenesis of HCC in the context of HCV infection involves several pivotal aspects. Unlike HBV, HCV is not recognized as a direct carcinogen, as it does not integrate into the host genome. However, an abundance of evidence indicates that chronic HCV infection represents a significant risk factor for HCC [10]. This chapter focuses on the analysis of various pathogenetic mechanisms that contribute to the development of HCC in patients with CHC. The division into different aspects of pathogenesis is implemented for didactic reasons, given that the diverse processes occurring during the development of HCC are interconnected and unfold concurrently.

### 2.1. Chronic Inflammation

Long-term inflammation of the liver is a key driver in the development of fibrosis, which involves the accumulation of extracellular matrix (ECM) proteins including collagen and the disruption of the normal liver architecture [11]. Prolonged inflammation is maintained through a variety of inflammatory mediators, which are also recognized as contributors to the process of carcinogenesis. Research findings in the field of basic medical sciences have demonstrated the significant role of inflammation during chronic HCV infection [12]. Some inflammation-inducing proteins could potentially serve as indicators of inflammation in patients with CHC [13]. Interleukin (IL)-6 and IL-8, C-X-C motif ligand (CXCL)-9, CXCL-10, CXCL-12, and macrophage migration inhibitory factor (MIF) have the potential to function as indicators for evaluating the advancement from chronic hepatitis to cirrhosis [14,15,16]. Transforming growth factors (TGF)-α and TGF-β significantly impact hepatocarcinogenesis [12,17,18]. Chronic liver damage-induced inflammation leads to TGF-α upregulation in the liver, driving regeneration, hepatocyte dysplasia, proliferation, and, ultimately, HCC development. TGF-β plays a crucial role in the later phase of inflammation, promoting tissue repair, inhibiting leukocyte activation, and affecting adhesion molecules on parenchymal cells. TGF-β counters proinflammatory cytokine effects, restraining processes like proliferation and differentiation. Ironically, cancer cells exploit these changes to their advantage. In the intricate process of carcinogenesis, malignant cells frequently weaken suppressive TGF-β signaling by altering receptor expression and co-opting the signaling pathway [19]. In the initial phases of cancer, TGF-β is perceived as a suppressor that triggers apoptosis, whereas in later stages, there is a shift towards promoting events such as epithelial-mesenchymal transition, invasion, metastasis, and angiogenesis [19,20]. Combined TGF-β and IL-6 exposure in human HCC cells attenuates IL-6-induced proproliferative effects by TGF-β, affecting IL-6R transcription, signal transducer and activator of transcription (STAT)-3 expression, nuclear localization, and p65 activation. SMAD-dependent TGF-β leads to epithelial–mesenchymal transition, losing polarity and acquiring invasive properties [12,17,18].

Tumor Necrosis Factor-α (TNF-α) itself is a potent proinflammatory cytokine [21]. TNF-α has the potential to induce liver damage, cirrhosis, and eventually facilitate the progression of HCC. Elevated production of TNF is linked to an escalation in the secretion of proinflammatory cytokines, the activation of proto-oncogenes, and various genes connected with cell proliferation, invasion, and the metastasis of cancer cells [22]. Research has indicated that signal nucleotide polymorphisms (SNPs) in the TNF-α gene, including −863 C/A, −857 C/T, −308 G/A, and −238 G/A, are linked to the risk of developing HCC. These SNPs located on the TNF-α promoter can influence the level of TNF-α expression and lead to increased and continuous TNF-α production and are associated with a heightened HCC risk [23]. A substantial correlation was observed between the TNF-a GG genotype, and the occurrence of HCC [21]. Moreover, TNF-α is also recognized for its role in inducing HCC by activating and differentiating hepatic progenitor cells through the chronic inflammatory pathway [12]. The significant influence of TNF-α on liver injury and prognostic outcomes was confirmed by the study conducted by Jing et al. They showed that elevated TNF-α levels stimulate the development of HCC by activating hepatic progenitor cells, while the absence of TNF-α restrains the activation and growth of these cells, thereby reducing the occurrence of tumors [24].

Mitochondria are recognized as the primary generator of reactive oxygen species (ROS) in CHC [25]. Chronic HCV infection triggers mitochondrial dysfunction, resulting in an overabundance of oxidative stress. HCV-induced oxidative stress contributes to extensive inflammation and the fibrosis progression, but also insulin resistance and carcinogenesis [26,27]. Furthermore, oxidative stress predisposes to damage in genomic and mtDNA, while also impairing DNA damage repair pathways [28]. It is interesting that the impact of oxidative stress seems to be more pronounced in CHC compared to autoimmune hepatitis or chronic hepatitis B [27]. Protective mechanisms against ROS encompass reducing agents like glutathione, oxidoreductases, and peroxidases. The transcription factor NF-E2-related factor 2 (Nrf2) encoded by the Nuclear factor erythroid 2-related factor 2 (*NFE2L2*) assumes a pivotal function in regulating cellular redox homeostasis through the regulation of various cytoprotective enzyme expressions [29,30]. As demonstrated in Nrft2-deficient mice, the absence of Nrf2 results in impaired liver regeneration, with oxidative-stress-mediated insulin/insulin-like growth factor resistance as an underlying mechanism [30]. A reduction in the expression of cytoprotective genes, which in turn causes an increase in ROS levels, ultimately triggers the activation of the c-Jun N-terminal kinase (JNK) pathway, which is known to be involved in carcinogenesis [29]. Furthermore, the activation of JNK results in the phosphorylation of IRS1/2 at Ser/Thr positions, undermining the insulin signaling pathways [25].

The development of HCC in the context of CHC involves tangled interplay among multiple proinflammatory cytokines (such as IL-6, TNF-α) and anti-inflammatory cytokines (like TGF-α and β), oxidative stress, various transcription factors, and their associated signaling pathways. An early diagnosis of HCC is of vital importance for both patient and doctor. Assessing cytokines that trigger gene transcription involved in HCC genesis could be a precise diagnostic instrument complementing imaging techniques [31].

### 2.2. Liver Fibrosis and Cirrhosis

Over the years, untreated CHC typically involves chronic inflammation, leading to fibrosis, followed by progression to cirrhosis in some cases. Ultimately, this sequence culminates in an increased risk of HCC development [32]. Around 20% of individuals with CHC experience the development of liver cirrhosis over a period of 20 to 30 years. Once cirrhosis sets in, the annual probability of HCC occurrence ranges from 1% to 4%, with the 5-year cumulative HCC risk being 30% in Japan and 17% in Western countries [10,33]. While most HCV-related cases of HCC develop in individuals with cirrhosis, there are instances where it can arise in the context of bridging fibrosis. Consequently, the present guidelines suggest HCC monitoring, which involves biannual liver ultrasound, combined with alpha-feto protein (AFP) testing for patients who have cirrhosis or F3 fibrosis [34,35]. From a histological standpoint, liver fibrosis encompasses the accumulation of ECM proteins, particularly collagen, forming complex structures within the liver tissue [36]. Among the main contributors to collagen production are hepatic stellate cells (HSCs) and fibroblasts. In healthy individuals, HSCs are perisinusoidal cells that store vitamin A and are capable of producing collagen types IV and VI in the case of liver injury. Oxidative stress arising from perturbed cellular redox balance triggers the commencement of liver fibrosis through the generation of ROS and reactive nitrogen species (RNS) [37]. ROS include different reactive molecules such as peroxide, superoxide, hydroxide, and singlet oxygen. HSCs are activated by ROS during HHC or other chronic liver diseases. Consequently, these cells undergo transformation into myofibroblasts and accumulate fibrillar collagens such as types I and III. Myofibroblasts play a role in the development of fibrosis while also contributing to processes such as regeneration, inflammation, angiogenesis, and stromal reactions during tumorigenesis [38]. HSCs are a primary source of α-SMA-expressing myofibroblasts in various experimental liver diseases. Consequently, cancer-associated fibroblasts (CAFs) are likely to originate from HSCs. Nevertheless, certain debates persist, and aside from HSCs, other factors could also be involved. CAFs are believed to have an impact on ECM composition, as the components of the ECM can influence the behaviors of cancer cells and stromal cells through direct and indirect interactions, leading to functional changes [39]. CAFs that reside within the tumor microenvironment (TME) of HCC have diverse functions, including the promotion of tumor growth, progression, metastasis, and angiogenesis [40]. Hypoxia and persistent inflammation in the liver contribute to the development of fibrosis and cirrhosis, while the disrupted liver architecture subsequently leads to angiogenesis. Angiogenesis represents one of the major pathological characteristics of liver cirrhosis. It contributes to the development of portosystemic collateral vessels and the occurrence of portal hypertension, as well as advancement from liver fibrosis to cirrhosis and HCC [14]. Hypoxia, oxidative stress, inflammation, and impaired endothelial function can potentially trigger the activation of proangiogenic factors. Vascular endothelial growth factor (VEGF), placental growth factor (PlGF), and platelet-derived growth factor (PDGF) induce significant neovascularization within the liver and mesenteric vascular region in the context of cirrhosis [41]. Angiopoietins (Angs) and their corresponding tyrosine kinase receptors are cytokines with proangiogenic properties that are upregulated in liver cirrhosis, impacting prognosis [42]. Furthermore, angiogenesis holds a crucial significance in the progression of HCC, VEGF, and Angs serving as pivotal instigators of angiogenesis in HCC [43]. Angiopoietin-1 and angiopoietin-2 were found to be elevated in individuals with HCC and exhibited a correlation with both tumor dedifferentiation and the density of blood vessels within the tumor [39].

Gaining insight into the cellular triggers and molecular mechanisms driving fibrogenesis in CHC and other chronic liver diseases could potentially contribute to impeding the transition to advanced fibrosis and cirrhosis, thus likely diminishing the risk of developing HCC [44].

### 2.3. Immune Response Dysfunction

In the cascade of HCC development during chronic HCV infection, special importance is attached to immune response dysfunction. The ability of HCV to affect various types of immune cells and the liver microenvironment and to manipulate the entire immune response in its favor is key to the development of persistent infection. This, in turn, leads to chronic inflammation and, in the further course, to the carcinogenesis of HCC [45]. Thus, HCV avoids immune surveillance and elimination not only by mutations of its own genome (primarily in hypervariable region 1 within E2, NS3, and NS5), but also by inhibiting both the innate and adaptive arms of the immune response. This inhibition includes dendritic cells (DC), macrophages (Kupffer cells), natural killer (NK) cells, and CD4+ and CD8+ T cells. Additionally, studies have shown that, apart from these cells, B cells and peripheral blood mononuclear cells (PBMCs) also serve as reservoirs for HCV [46]. HCV proteins play a significant role in this dysfunction of the immune system. The NS3/4A serine protease interferes with retinoic acid-inducible gene-I (RIG-I) and toll-like receptor (TLR) 3 signaling, thereby affecting endogenous interferon (IFN) production [47]. Core protein degrades STAT-1, which inhibits its activation, and also inhibits the binding of interferon-stimulated gene factor 3 (ISGF3) to the IFN-stimulated response elements (IRES). Also, together with the E1 protein, it adversely affects DC maturation and their secretion of IL-12 and IL-2, which are key in the activation and proliferation of T cells [48]. Additionally, core protein affects the production of inflammatory cytokines IL-1 and IL-6, through interaction with gC1qR on macrophages. E2 protein, binding to CD81, affects the cytolytic function of NK cells, and NS4A/B affects the function of CD8+ T cells through the production of protein that can block HLA class I expression on surface of infected cells [49,50]. The immunological inhibitory effect on the microenvironment is also manifested by the induction of apoptosis of mature DC independently of p53. Some studies indicate that it is Fas-mediated apoptosis, and the importance of upregulation of cellular FADD-like interleukin-1β converting enzyme (FLICE) is highlighted [46,51].

All of the mentioned disorders of immune mechanisms, which include components of both innate and adaptive immunity, are related to carcinogenesis and the development of HCC. Thus, the inhibition of the function of NK cells includes their reduced antitumor activity in the microenvironment of HCC in the direct killing of tumor cells. The reason for this phenomenon is a decrease in the function of their activating receptors. It is considered that one of the most important is the NK group 2D (NKGD2D) receptor, through which NK cells recognize major histocompatibility complex class I chain associated molecules (MIC) A/B. The disruption of this interaction is crucial for early hepatocarcinogenesis [52]. NK cells with a reduced NKG2D profile are precisely those found in HCC tumor tissue compared to normal liver tissue [53]. On the other hand, the cytokine secretion of these cells is also disturbed, with a greater secretion of IFN-α and a reduced secretion of IFN-γ. This functional dichotomy is significant in hepatocarcinogenesis [54].

In the liver microenvironment, the production of IFN type I and IFN type III is insufficient during chronic HCV infection. The type I IFN family, which consists of multi-gene cytokine family encoding 13 partially homologous IFN-α subtypes, a single IFN-β, and several other poorly defined single gene products, has been shown to have a significant effect on tumor tissue, as well as on immune and endothelial cells [55]. By their effect, they directly affect the cell cycle, causing the death of HCC tumor cells, and they also have an effect on immunogenicity via MHC-I and CD8+ T cells, as well as other cells of the immune system such as DC, NK cells, neutrophils, regulatory T (Treg) cells, and myeloid-derived suppressor cells (MDSCs) [56]. The function of IFN type III, including IFN-λs 1–4 in humans, is disturbed and generally repressed These interferons achieve their function through the specific receptor chain IFN-λR1, and IL-10R2, a receptor chain shared by IL-10 cytokine family members, IL-10, IL-22, and IL-26. It has been shown that they also have an antitumor effect, inducing apoptosis through a direct effect on malignant cells [57]. Particularly, experiments on a mouse model showed that the application of IFN-λ has an important antiproliferative effect on malignant hepatocellular carcinoma cells [57].

The dysfunction of the CD8+ T cells is also an important mechanism in the development, and particularly in the recurrence, of HCC. CD8+ T cells specific to tumor antigens, especially tumor-associated antigen (TAA), are the most important antitumor effector cells, exhibiting both cytotoxic and noncytolytic antiviral functions [58]. During chronic HCV infection, continuous antigen stimulation leads to CD8+ T cells exhaustion, resulting in two subsets: TCF-1 + CD127 + PD-1+ memory-like cells and PD-1 high Eomeshi CD127 cells. Such CD8+ T cells, with reduced function, combined with negative effects of the tumor microenvironment, fail to effectively destroy HCC tumor cells [59]. A special problem is the fact that their recovery does not occur even after successful antiviral therapy, either PEG IFN-α-based or DAA, with achievement of SVR. 

A significant disorder in the functioning of immune cells of the liver was also detected in intrahepatic macrophages and mucosal-associated invariant T cells (MAIT cells). Intrahepatic activated macrophages increase production of IL-1β due to NOD-, LRR-, and pyrin domain-containing protein 3 (NLRP3) stimulation. On the other hand, MAIT cells that can be activated by IFN type I, IL-12, IL-15, and IL-18 are immune-exhausted, due to constant antigenic stimulation. Upregulation of exhaustion markers such as PD-1, CTLA-4, and Tim-3 causes decreased production of IFN-γ [60,61]. An increase in the frequency of CD4+CD25+FoxP3+ regulatory T cells, as well as MDSCs, is also important. A special problem with the mentioned immune changes is that they do not normalize after antiviral treatment. Additionally, elevated levels of serum cytokines, such as IL-3, IL-21, IL-22, tumor necrosis factor-like weak inducer of apoptosis (TWEAK), a proliferation-inducing ligand (APRL), VEGF, etc., are registered in patients who develop HCC after successful antiviral therapy, compared to those who do not develop HCC [62]. 

All the mentioned dysfunctions in the immune response, as well as potentially many others yet to be discovered, combined with other pathogenic mechanisms of carcinogenesis that are discussed in this article, are responsible for both the occurrence and recurrence of HCC. This can happen even after successful antiviral therapy for chronic HCV infection.

### 2.4. Oncogenic Signaling Pathways

The main points in molecular carcinogenesis are mutations in oncogenes and tumor suppressor genes, followed by genetic DNA instability. Most of the evidence from basic research implies that mainly the HCV core protein and the NS3 and NS5A may have an active role in the development and progression of HCV-associated liver disease and HCC. HCV core, NS3, and NS5A overexpression enhances cellular proliferation, transformation, and tumor formation in transgenic mice, suggesting a direct role in triggering carcinogenic molecular pathways [63,64,65,66]. Variable gene mutations were found to be linked to hepatocarcinogenesis [67]. The most important and first identified gene was tumor suppressor gene *p53* [68]. The HCV core protein inhibits *p53* as well as *p73* [69,70]. Kao et al. analyzed the in vitro and in vivo interactions between HCV core protein and p53 which are colocalized in subnuclear granular structures and the perinuclear area and showed that *p53* and HCV core protein form a specific complex [69]. The authors further reached contradicted results of the interaction between HCV core protein and p53. Namely, it was demonstrated that low concentrations of HCV core protein stimulate the p53 activity, while high levels suppress it [69]. The results of the research finally indicate that the interaction between HCV core protein and *p53* pathway occurs via at least three mechanisms: physical complex forming, modulation of *p53* gene regulatory activity, and post-translational modification, such as acetylation and phosphorylation. The HCV core protein induces hyperacetylation of *p53* at Lys373 and Lys382, leading to the increase in *p53* DNA-binding activity. Furthermore, HCV core protein can alter biphasically the Ser15 phosphorylation. In cells expressing low level of HCV core protein, it causes the enhancement of Ser15 phosphorylation of *p53*, and in cells expressing high levels of HCV core protein, it causes the suppression of Ser15 phosphorylation of *p53* [69]. NS3 and NS5A inhibit *p53* by promoting its relocation from nucleus to cytoplasm [71,72]. The results of Deng et al. showed that *p53* expression levels were not altered significantly by NS3-N (N terminal of NS3) and Kwun et al. also demonstrated that neither *p53* mRNA nor protein levels were downregulated by NS3 [71,73]. According to all mentioned, it is possible that NS3-N inhibits *p53* function by interacting with it physically [73].

NS5A also physically associates with *p53* both in vitro and in vivo and sequesters *p53* in the perinuclear membrane. Subsequently, a decrease in *p53* in the nucleus may lead to the downregulation of the *p53*-mediated gene expression required for normal cell growth [72]. Another tumor suppressor gene affected by HCV proteins is retinoblastoma1 (*RB1*), which is inhibited by HCV core and NS5B proteins [74]. Retinoblastoma protein controls cell proliferation predominantly by suppressing E2f activation. NS5B polymerase is responsible for posttranscriptional downregulation of RB [74,75]. Namely, the results of Munakata et al. revealed that RB mRNA levels were not altered by the presence of NS5B [75]. The authors further demonstrated that NS5B interacts with RB perinuclearly, forming a stable complex, and targets it for degradation before its transport to nucleus [75]. On the contrary, HCV core protein seems to decrease the level of pRB (the product of Rb gene) by downregulation of the RB mRNA [75]. Mileo et al. found that the HCV core protein expression downregulated pRb2/p130 protein and mRNA levels in HuH-7-CORE cells by inducing promoter hypermethylation [76]. Kim et al. also reported that the HCV core protein suppressed the expression of Rb promoters in HepG2 cells [77].

The activation of telomerase is associated with certain types of cancer, and according to the literature data, it was found in around 85% of HCC tissues [78]. Telomerase is an enzyme that prevents shortening of telomeres, which is a normal process during cell life cycle. Prevention of telomere shortening leads to uncontrolled cell proliferation. Chen et al. indicated that TERT (telomerase reverse transcriptase) promoter mutations frequently occur in HCV-related HCC [79]. The study also demonstrated that absence of hepatitis B infection was significantly associated with the TERT promoter mutation [80]. TERT is re-expressed not only in HCC cells, but also in some premalignant lesions like low- and high-grade dysplastic nodules. Nault et al. reported TERT promoter mutations in 6% of low-grade and in 19% of high-grade dysplastic nodules [81,82]

Release of ROS remains a critical part for HCV-induced HCC. It was suggested that core expression increases the production of ROS, which results in an impaired mitochondrial β-oxidation [83]. NS5A also has a role in the production of ROS. NS5A overexpression promotes calcium release from endoplasmatic reticulum (ER), leading to its quick absorption by mitochondria and subsequent elevated production of ROS [84,85]. ROS contributes to carcinogenesis by promoting DNA damage and gene mutation as well as by altering different cell signaling pathways [86]. ROS affect *Sp1* (specific protein 1) gene, **MDM2** (Mouse double minute 2), *p53*, and *DHCR 24* (3β-hydroxysteroid-Δ24 reductase) [86]. The Sp-family of proteins regulates the expression of genes that play crucial roles in cell proliferation and metastasis of various tumors. High levels of Sp1 protein are considered a negative prognostic factor. The *MDM2* gene encodes a nuclear-localized E3 ubiquitin ligase. The encoded protein can promote tumor formation by targeting tumor suppressor proteins, such as *p53*, for proteasomal degradation. Wu et al. demonstrated that *DHCR24* was involved in cells invasion and migration by modulating cholesterol biosynthesis and lipid rafts formation in HCC [87]. Furthermore, high expression of *DHCR24* in HCC patients was markedly correlated with poor clinical outcome [87]. ROS affect the occurrence, proliferation, metastasis, and angiogenesis of tumors [86].

The HCV core protein can modulate several signaling pathways involved in cell cycle regulation, cell growth, proliferation, apoptosis, oxidative stress, and lipid metabolism [69,77,88,89,90,91,92]. The dysregulation of these signaling pathways, such as TGF-β, VEGF, Wnt/β-catenin (WNT), cyclooxygenase-2 (COX-2), and peroxisome proliferator-activated receptor α (PPARα), favors the development of HCC [88,89,90,91,92,93,94,95,96,97,98,99]. HCV proteins interfere with TGF-β (TGF-β1 isoform), leading to the transition of its activity from tumor suppressor to fibrogenic, resulting in increased risk for HCC. TGF-β plays a dual role in HCC formation, acting as a tumor suppressor in the early stages and a tumor promoter later on, by stimulating the production of antiapoptotic genes [89,90,91,92,93,94,95]. The HCV core protein can induce TGF-β1 promoter activity [89,90]. Taniguchi et al. showed that TGF-β1 mRNA expression is increased by the HCV core protein [89]. In other studies, it was revealed that the core protein was able to shift TGF-β responses from tumor suppressor to tumor promotor by decreasing hepatocyte apoptosis and increasing epithelial–mesenchymal transition (EMT) [91,92]. Moreover, the HCV core protein activated the latent form of TGFβ through increased thrombospondin expression [92]. It has also been shown that HCV-induced transcription factors such as AP-1 (activator protein 1), Sp1, NF-κB (Nuclear factor kappa B subunit 1), EGR-1, USF (upstream stimulatory factor), and STAT-3 (signal transducer and activator of transcription) can activate the TGF-β1 promoter [100]. Choi et al. showed that the HCV NS5A protein specifically interacts with TGF-*β*R-I in vitro and in vivo [100]. NS5A blocks TGF-*β*-mediated nuclear translocation and phosphorylation of Smad2 [101]. The Hepatitis C virus NS3 protein enhances HCC cell invasion, causing downregulation of PPM1A (protein phosphatase magnesium-dependent 1A) by promoting its ubiquitination and degradation [102]. PPM1A functions as a Smad phosphatase to terminate TGF beta signaling [103]. PPM1A is as an important tumor suppressor that can block several tumor-centric signaling pathways through protein dephosphorylation. Furthermore, TGF-β1 expression gradually decreases following viral clearance [95].

By upregulation of VEGF and COX-2, and by activating matrix metalloproteinases, the HCV core protein stimulates angiogenesis [96]. Neoangiogenesis is essential for tumor growth, as well as for spread of malignant cells and forming of intra- and extrahepatic metastases.

The hepatitis C virus core protein triggers hepatic angiogenesis by a mechanism including multiple pathways. In a study by Shao et al. among 131 tissue samples from HCC patients, HCV-related HCC revealed stronger VEGF expression compared to hepatitis B virus-related HCC [97]. The results imply that increased VEGF expression through AP-1 activation is a crucial mechanism underlying the proangiogenic activity of the HCV core protein in HCC cells [97]. The authors confirmed that HCV core protein expression potentiated the in vivo angiogenesis activity of HCC cells. Namely, two stable clones with HCV core protein expression, HuH7-core-high and HuH7-core-low, were established, as well as another clone without the expression of the HCV core protein, which served as a control [97]. The results of the analysis showed that both HuH7-core-high and HuH7-core-low cells induced more potent angiogenesis than cells without HCV core expression, and this potentiation of the proangiogenic effect was dose-dependent [97]. The HCV core protein also increases VEGF expression through increased activity of the androgen receptor signaling pathway [98]. Furthermore, Hassan et al. reported that the HCV core protein induces VEGF expression mediated by JNK, *p38*, and *ERK* signaling pathways [99]. HCV promotes the creation of new vasculature through the core-mediated activation of hypoxia-inducible factor 1 alpha (HIF-1), which results in enhanced VEGF expression [99].

**Figure 1:**
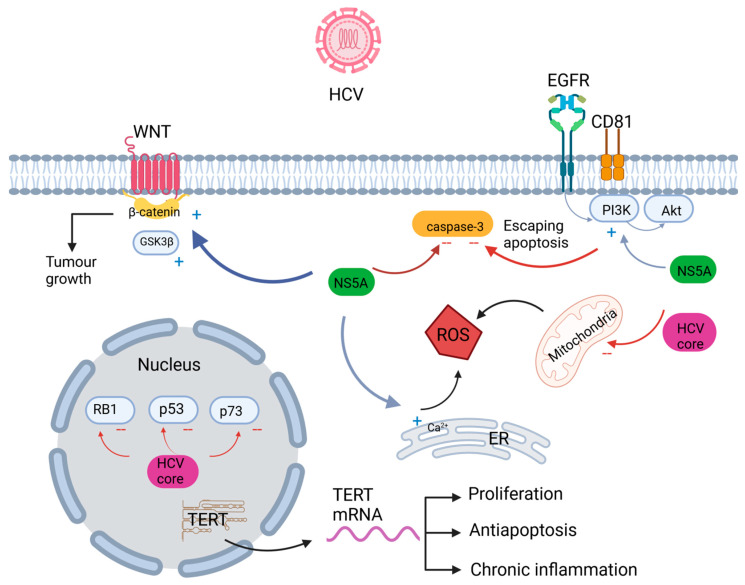
Molecular pathways in hepatitis C virus carcinogenesis (created using BioRender.com (accessed on 23 September 2023)). HCV: hepatitis C virus; EGFR: epidermal growth factor receptor; WNT: wingless-related integration site; PI3K: Phosphatidylinositol 3-kinase; Akt: AKT serine/threonine kinase; NS5A: Nonstructural protein 5A; ROS: reactive oxygen species; RB1: Retinoblastoma 1; ER: endoplasmatic reticulum.

The WNT/β-catenin pathway regulates multiple cellular processes that are involved in the development of HCC [104,105,106,107]. It was also shown that NS5A is involved in the activation of PI3K/AKT and beta-catenin/WNT pathways and escaping apoptosis by inhibition of caspase 3 [108]. Mutations in the oncogene catenin beta 1 (CTNNB1) were found in more than 30% of patients with HCV-induced HCC [109,110]. This gene is responsible for beta-catenin coding. NS5A and HCV core protein were found to activate beta-catenin when overexpressed in cell culture through an indirect mechanism which includes phosphorylation and inactivation of GSK 3beta, but NS5A can also activate beta-catenin through a direct mechanism [111]. It is speculated that dysregulation of WNT signaling pathways is not sufficient to cause malignant transformation of hepatocytes, but might be responsible for tumor growth [112]. It was also shown that activating mutations in CTNNB1 correlates with a low response to immune checkpoint inhibitors (ICIs) monotherapy in advanced HCC patients [113]. The activation of WNT/β-Catenin leads to ICIs resistance by impairing antigen-specific T cell-mediated antitumor immunity [114,115,116]. Considering the abovementioned points, the WNT/-catenin pathway has shown promise as a novel molecular therapy target. Different oncogenic signaling pathways are schematically shown in Figure 1 (Adapted from [116]).

### 2.5. Epigenetic Changes

Another important mechanism through which HCV stimulates hepatocarcinogenesis relies on epigenetic changes in the genome. Epigenetic dysregulation can result in the suppression of tumor suppressor gene expression and the overexpression of certain oncogenes, resulting in carcinogenesis. Epigenetic mechanisms include DNA methylation, post-translational histone modifications, chromatin remodeling, and noncoding RNA-mediated gene silencing.

HCV infection causes HCC primarily through dysregulation of cell apoptosis and development [117].

DNA hypermethylation of the promoter regions of certain tumor suppressor genes inhibits the expression of these genes. There is a long list of numerous genes which are targeted by epigenetic changes in HCC related to HCV infection. Some of the tumor suppressor genes that are downregulated in HCV-related HCC include RAS protein activator like 1 (*RASAL1*), *RASSF1A (RAS association domain family protein 1A), EGL nine homolog 3 (EGLN3), CUB* and *Sushi Multiple Domains 1 (CSMD1), cyclin-dependent kinase inhibitor 2A (CDKN2A), BCL 6 corepressor like 1 (BCORL1), secreted frizzled-related protein 1 (SFRP1), zinc finger protein (ZNF) 382, runt-related transcription factor 3 (RUNX3), lysyl oxidase (LOX), RB1, P73, APC (adenomatous polyposis coli), and mismatch repair system genes* [117,118,119,120,121,122,123,124,125,126,127,128,129,130,131,132,133,134,135,136,137,138,139,140,141,142]. *APC* hypermethylation is present in over 80% of human HCC [142]. The abnormal methylation of the mismatch repair system may occur in up to 75% of HCC, and is also present in cirrhotic liver, suggesting that this aberration may represent an early step of hepatocarcinogenesis [139,140,141]. Zhang et al. reported that the promoter hypermethylation of *RASSF1A* was present in 85% of HCC patients [138]. Methylation of the *p16INK4A* gene, a potent cell checkpoint regulator, was found in up to 85% of the cases studied [143].

Some of the oncogenic genes that are upregulated by demethylation in HCV infection are *zinc finger* and *BTB* domain containing 16 *(ZBTB16), orthodenticle homeobox 2 (OTX2), insulin-like growth factor 1 receptor (IGF1R), synuclein gamma (SNCG), forkhead box protein A1 (FOXA1), hepatocyte nuclear factor 4 alpha (HNF4A), and CCAAT enhancer binding protein alpha (CEBPA*) [123,143,144,145,146]. *SNCG* is abnormally expressed in 65.7% of liver cancer and varies according to the stage of the disease. Importantly, all patients with metastatic disease contained the demethylated *SNCG* gene in their primary tumors [123].

The Gene Ontology analysis was conducted and showed that tumor suppressor and protooncogenes affected by hyper- or hypomethylation are engaged in different cell processes. Tumor suppressor genes are hypermethylated which leads to their down-expression, while oncogene genes are hypomethylated and subsequently overexpressed. The results of the gene ontology analysis showed that the majority of tumor suppressor or proto-oncogenes that were affected by hyper- or hypomethylation were involved in the process of cell apoptosis and cell development (seven genes involved in each process), while five and three genes were involved in the regulation of myeloid and lymphocyte cell differentiation, respectively [117].

HCV-related HCC has been linked to repetitive elements (REs) in noncoding sections of the genome. Although the precise molecular mechanisms behind liver cancer development remain unknown, it has been revealed that DNA methylation of these locus-specific REs is downregulated in HCV-induced HCC [147]. Repetitive elements including long interspersed element-1 (LINE-1) and Alu element (Alu) activate oncogenic pathways in HCC [148,149]. LINE-1 and Alu represent the two most abundant types of RE sequences that can mobilize in the human genome, causing genetic instability [148,149]. The methylation and expression analysis performed by Zheng et al. showed a functional role of hypomethylation in 12 HCV-HCC dmREs that downregulated their proximal genes, some of which were associated with HCC [147]. RE methylation suppresses RE mobility, leading to the stabilization of chromatin. When adequate methylation is perturbed, it can lead to different diseases, including cancer.

Furthermore, DNA methyl transferases (DNMTs) regulate gene silence caused by DNA methylation. HCV infection has an effect on DNMT expression as well. Viral proteins, core and NS5A, can be detected in the nucleus and hence near to host DNA [150,151,152]. The HCV core protein significantly boosts the expression of DNA methyltransferase (DNMT)-1 and histone deacetylase (HDAC)-1 and causes downregulation of tumor suppressor gene expression by DNA-methylation of cytosine-phospho-guanine (CpG) dinucleotides in regulatory gene elements [120].

Different HCV genotypes have been shown to influence DNMT mRNA and protein expression. Siddiqui et al. found that mRNA and protein expression levels of DNMT1, 3a, and 3b were upregulated in patients infected with HCV genotype 1b and 3a. On the other hand, DNMT3b mRNA levels were not changed in genotypes 2a, 3, and 4, but were upregulated at the protein level by genotypes 1b, 2a, and 3a [153].

Histone modifications are another group of epigenetic mechanisms that play important roles in regulating gene expression and changes in chromatin structure. DNA is packed into chromatin with the help of histone protein octamers. Post-translational histone modifications include acetylation, methylation, phosphorylation, and ubiquitination. Histone acetylation, which is dynamically regulated by histone acetyltransferases (HATs) and histone deacetylases (HDACs), is common in the development of HCV-infected HCC. Examples of significant histone acetylation are histone H3 acetylated on lysine 9 (H3K9Ac), histone 3 acetylated on lysine 27 (H3K27Ac), H2A acetylated on lysine 5 (H2AK5ac), and H3 acetylated on lysine 14 (H3K14Ac) [154].

A study by Hamdane et al. showed that chronic HCV infection induces significant epigenetic H3K27ac changes on genes that belong to pathways related to TNF-α, inflammatory response, and IL-2 and signal transducer and activator of transcription 5 signaling [155]. An interesting point is that since epigenetic regulation of gene expression is considered as a lasting “epigenetic memory”, it may suggest that HCV-induced changes persist even after viral cure [156,157]. The abovementioned study by Hamdane et al. also addressed this issue and demonstrated that virus-induced modifications of histone mark H3K27ac persist in human liver cells after cure with direct antiviral agents (DAAs) in HCV-infected patients [155]. Furthermore, it was shown that persistent epigenetic changes are associated with liver carcinogenesis after cure. The study further analyzed the association of the stage of liver fibrosis at the moment of HCV elimination with the persistence of epigenetic modifications [155]. The analysis showed that H3K27ac changes observed in HCV-infected patients were partly reversed in cured patients with stage F2–3 fibrosis [155]. In contrast, in DAA-cured patients with advanced liver fibrosis, the HCV-induced H3K27ac changes largely persisted. Namely, almost all modified genes (96.6%, 5140 of 5318 genes) present in HCV infected persons persisted in patients with F4 fibrosis after virological cure. In patients with F2-F3 fibrosis, 2259 of 5318 (42.5%) remained after the achievement of sustained virological response [155].

Noncoding RNAs (ncRNAs) also play an important role in the regulation of various cancers, including HCC [158,159]. Two types of ncRNAs, microRNAs and long noncoding RNAs (lncRNAs), have been the focus of recent research. LncRNAs are more than 200 nucleotides in length and participate in various biological processes and human disease. In the context of HCV-related HCC, two lncRNAs, HIF and PAR5, have been associated with HCV-induced HCC [160,161]. Additionally, seven other lncRNAs, including LINC01419, BC014579, AK021443, RP11-401P9.4, RP11-304 L19.5, CTB-167B5.2, and AF070632, exhibit differential expression and have the potential to serve as biomarkers for the early detection of HCC [162].

### 2.6. MicroRNA Dysregulation

MicroRNAs (miRNAs) are small noncoding RNA molecules that regulate gene expression by binding to the messenger RNA (mRNA) and degrading it or inhibiting its translation into protein. Today, it is well known that dysregulated miRNAs play a big role in the development and progression of HCC in HCV-positive patients. They participate in various cellular processes, including cell proliferation, apoptosis, angiogenesis, and metastasis, via multiple pathways, such as immune response, lipid metabolism, and cell-cycle pathways [163,164,165]. MicroRNA dysregulation in HCC is a complex phenomenon that involves both upregulation and downregulation of specific miRNAs. Many studies have identified certain miRNAs as key players in the interaction between the virus and the host, as well as in the pathogenetic processes during HCV infection and virus replication, and also in the development of HCC [164,165,166]. Many miRNAs included in this processes are increased, such as miR-21, miR-221, and miR-222, in contrast to miR-122a, miR-145, miR-199a, and miR-223, which are decreased [163,164,167,168,169]. There are two types of miRNAs: oncogenic miRNAs (oncomirs) and tumor suppressor miRNAs [163]. The expression levels of miR-21 are significantly upregulated [163,170,171,172,173,174,175,176,177,178]. Secreted miR-21 contributes to cancer progression affecting neighboring hepatic stellate cells and HCC cells. Its secretion leads to the activation of prosurvival pathway PDK1/AKT in hepatic stellate cells, and induces the secretion of factors that support angiogenesis in HCC cells, such as VEGF, basic fibroblast growth factor, MMP2, MMP9, and TGF-β [163,164,165,166,167,168,169,170,171,172,173,174,175,176,177,178,179]. Shrivastava et al. evaluated the expression of many miRNAs among HCV-positive patients with HCC. They observed elevated expression of three miRNAs, miR-625, miR-532, and miR-618, in 56%, 62.5%, and 72% of HCC-post HCV positive patients, respectively. Additionally, miR-516-5p and miR-650 were found to be downregulated in 50% and 72% of HCC-post HCV-positive patients, respectively. Furthermore, miR-155 expression levels were markedly increased in patients with CHC. Increased levels of this miRNA promote hepatocyte proliferation and tumor genesis in two ways. One way is through the activation of the β-catenin pathway, leading to increased expression of various genes, such as *cyclin D1*, *c-myc*, and *survivin*. The other way involves reducing the expression of adenomatous polyposis coli. Also, two other miRNAs promote tumor genesis: miR-141 and miR-491. miR-141 promotes tumor genesis by inhibiting the expression of the tumor suppressor gene, *DLC-1* (a Rho GTPase-activating protein), and miR-491 by targeting genes involved in the phosphoinositol-3 kinase-Akt pro survival pathway, such as *PTEN (Phosphatase and Tensin Homolog*), which is a negative regulator of this pathway. By targeting *PTEN*, miR-491 can indirectly enhance PI3/AKT signaling, thereby promoting cell proliferation and survival [165].

Varnholt et al. demonstrated that miR-122, miR-100, and miR10a are overexpressed, whereas miR-198 and miR-145 are up to fivefold downregulated in hepatic tumors compared to normal liver tissues. miR-122 was predominantly expressed in hepatocytes, constituting approximately 70%, of total miRNAs in the liver [164,180,181]. Observed increased expression of miR-122 in the large sample set could potentially lead to the increased tumor growth, contrary to previous studies that showed downregulation of miR-122 in non-HCV HCC cells [164,182]. The other two miRNAs, miR-221 and miR-222, are important for the development of HCC and its invasion, and they also serve as therapeutic targets [182,183]. Fu et al. discovered that higher expression of miR-221 promotes the migration of HCC cells by targeting gene plant homeo domain finger 2 183]. Wong et al. demonstrated that the expression of miR-222 is increased in HCC cells and that there is a connection between this heightened expression and tumor progression [182].

## 3. Future Perspectives

The mechanisms of carcinogenesis in patients with CHC and other CLDs offer new opportunities for cancer prevention, early detection, and treatment. Developing precise molecular techniques to target individual cytokines and influence their activity represents a potential avenue for both prevention and therapy.

Clinical trials involving cytokines or cytokine antagonists demonstrated limited therapeutic efficacy, probably because most trials included patients with advanced-stage diseases. This scenario might not be the most suitable context for cytokine-based therapy. Emerging strategies do not involve using cytokines as monotherapy, but, rather, utilizing them to amplify the effects or mitigate the negative impacts of alternative cancer therapies in the early stage of the disease. This approach should be based on the specific cytokine profile of each tumor [184].

Attempts of cancer therapy focused on oxidative stress have already been described. While many antioxidant compounds as therapeutics have shown promise in preclinical investigations, their performance in clinical trials has been unsatisfactory. A more comprehensive investigation of the ways that antioxidants function, along with their specific contexts of efficacy, could offer a solution to enhance their pharmacological effectiveness [185].

Further research is needed regarding the mechanisms of immune response dysfunction during the development and treatment of HCC, both in the microenvironment of the liver and in individual components of immunity. This research is necessary to facilitate their correction during treatment. Currently, immune checkpoint inhibitors such as atezolizumab, nivolumab, and pembrolizumab are in regular therapeutic use. However, there are other aspects of the immune system that need to be targeted to prevent and treat HCC. Therefore, the use of anti-PD-1/PD-L1 agents could invigorate exhausted T cells and restore their cytotoxicity against cancer cells, among other potential benefits.

In addressing various oncogenic signaling pathways, the primary focus should be on preventing their overexpression. Tumor neoangiogenesis is one of the potential targets for molecular therapy. Therefore, understanding the molecular basis of HCV-mediated hepatic angiogenesis will assist in developing therapeutic strategies for the prevention and treatment of HCV-mediated angiogenesis. The RAF/MEK/ERK signal transduction pathway is among the suggested suitable targets for antiangiogenic drugs [94,186,187].

The safety and immunogenicity of an hTERT-derived peptide (hTERT461) as a vaccine has been investigated in an in vivo study enrolling 14 HCC patients [188]. The vaccination induced hTERT-specific immunity in 71.4% of patients [188].

Because many epigenetic pathways have been shown to play important roles in HCC pathogenesis, they have also become interesting targets for cancer therapy. Furthermore, epigenetic changes were also recognized as one of the mechanisms of acquired drug resistance. Considering that unlike genetic mutations, epigenetic alterations can be modulated by pharmaceuticals, interventions directed towards these modifications could also regain the effectiveness of existing anticancer drugs like ICIs [189]. Epigenetic drugs not only have a direct effect on tumors but also have the potential to remodel the suppressed tumor microenvironment (TME) and synergistically improve ICI efficacy [189,190,191]. Initial therapy of the TEM using epigenetic drugs prior to immunotherapy may help transform immune cells toward cancer-fighting subtypes.

The first epigenetic medications which were investigated as an alternate strategy to cancer treatment were small molecule inhibitors of DNMT. Azacytidine and decitabine were among the first that were shown to resensitize resistant tumor cells to sorafenib [192,193,194]. Despite the promising preclinical data, these DNMT inhibitors generally have short half-lives, which significantly reduces their efficacy in vivo [195]. In order to overcome these limitations, second-generation DNMT inhibitors have been developed, like guadecitabine (SGI-110).

miRNAs could help identify early stages of HCC. They also have the potential to predict disease progression, thus modulating its expression and activity, offering a promising avenue for developing novel therapeutic strategies. miRNA profiling could contribute to the identification of molecular subtypes of HCC, enabling more targeted and effective treatments tailored to the specific characteristics of each patient’s disease. As miRNAs play a role in the metastatic processes of HCC, further research could elucidate the specific miRNAs involved in these processes and lead to the development of therapies targeting metastasis.

## 4. Conclusions

The authors acknowledge that this review cannot fully address all facets of carcinogenesis in patients with HCV infection. However, they believe it provides a valuable starting point for understanding the diverse factors contributing to the emergence of HCC. Not too long ago, HCV infection posed a significant treatment challenge. The introduction of DAA has significantly altered the prognosis of these patients and enabled ambitious goals, such as the pursuit of controlling hepatitis as a public health concern. Despite the increasing number of patients effectively treated with DAA, the risk for HCC development persists among individuals with cirrhosis, as well as in those who have concurrent comorbidities (alcohol, metabolic syndrome, NAFLD/NASH, HIV comorbidity, etc.). The development of novel tumor markers (e.g., cytokines-as IL-6, TNF-α, TGF-α and β, reliable markers for CAFs, and certain miRNAs) superior to the existing AFP could enable early detection of HCC, thereby increasing the potential for successful treatment and improved survival rates. Furthermore, understanding the nature of complex molecular processes involved in the development of HCC would open new possibilities for treatment and the implementation of precise and personalized medicine for each patient with HCC in the context of HCV infection, and other chronic liver diseases.
